# Calcineurin A/TAX-6 signaling inhibits noxious heat-evoked nuclear accumulation of CMK-1 in the FLP thermo-nociceptive neurons of *Caenorhabditis elegans*

**DOI:** 10.17912/micropub.biology.001918

**Published:** 2026-01-13

**Authors:** Domenica Ippolito, Martina Rudgalvyte, Dominique A. Glauser

**Affiliations:** 1 Dept. Biology, University of Fribourg, Chemin du Musée 10, 1700 Fribourg, Switzerland

## Abstract

Calcium signaling is central to neural plasticity. In *C. elegans* , the CaM kinase I/ CMK-1 and the phosphatase Calcineurin A/ TAX-6 antagonistically regulate thermo-nociceptive behavior via partially overlapping neuronal circuits. Following prolonged exposure to noxious temperature (90 min, 28°C) CMK-1 localization shifts from cytoplasmic to nuclear in FLP neurons, which reduces behavioral responsiveness. We examined whether Calcineurin A/ TAX-6 influences this process. Using CMK-1 ::mNeonGreen, we found that Calcineurin A/ TAX-6 activation blocks, while its loss slightly enhances, CMK-1 nuclear translocation at 28°C. These results reveal that Calcineurin A/ TAX-6 signaling negatively regulates CMK-1 nuclear accumulation, providing a potential mechanism for opposing modulation of thermo-nociceptive plasticity.

**Figure 1. Calcineurin A/TAX-6 signaling inhibits noxious heat-evoked nuclear accumulation of CMK-1 in FLP neurons:**
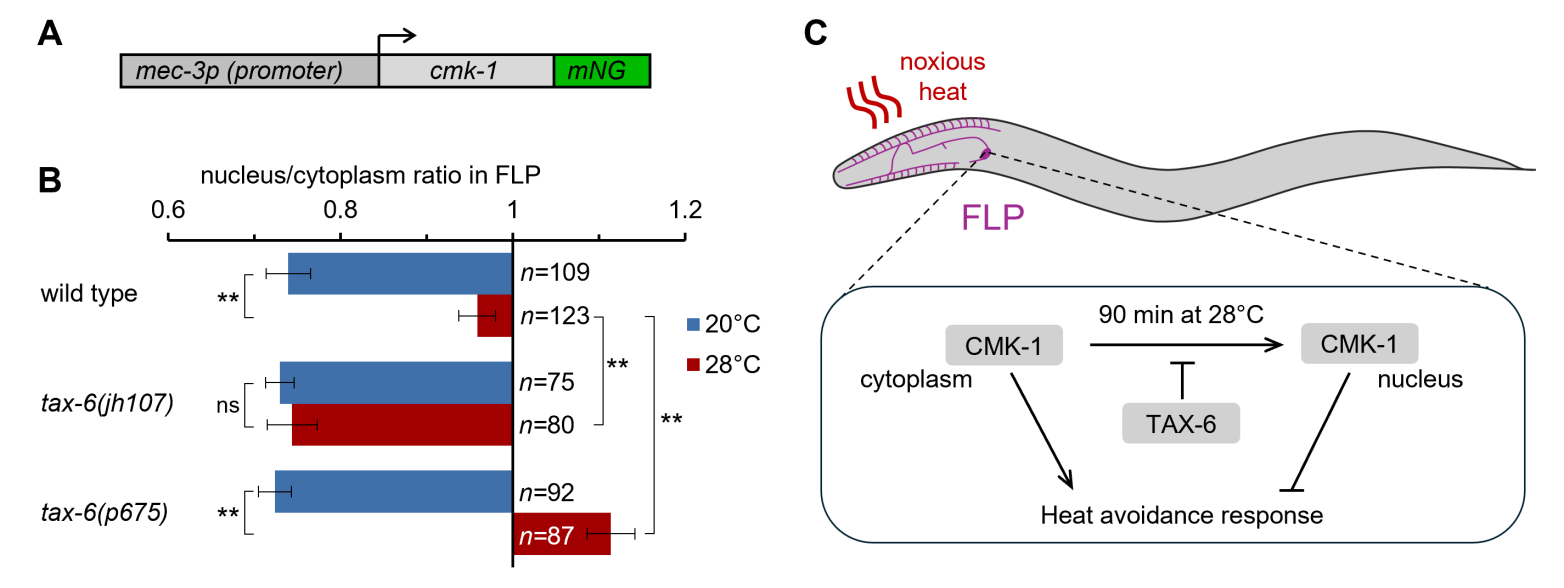
**A** : Schematic of the transgene used to express CMK-1 ::mNeonGreen (mNG) reporter in FLP neurons. **B:** Average nuclear/cytoplasmic fluorescent signal ratio (± SEM) of CMK-1 reporter in FLP, showing heat-evoked nuclear translocation of wild type CMK-1 after 90 min at 28°C compared with the control at 20°C, as well as the impact of the indicated *tax-6* mutations. *tax-6 ( jh107 )* is a gain-of-function allele. *tax-6 ( p675 )* is a loss-of-function allele. A two-way ANOVA revealed a significant *Temperature* × *Genotype* interaction effect (F₂,₅₆₀ = 33.2, p < 0.001). **, *p* <.001 by Bonferroni post hoc tests; ns, not significant ( *p=* .652). The numbers of neurons scored in each condition are indicated in the figure (n). **C** : Visual summary of a model in which TAX-6 activity inhibits the noxious heat-evoked nuclear accumulation of CMK-1 in the FLP thermo-nociceptive neurons of *C. elegans* in order to promote heat avoidance.

## Description

Calcium signaling, including the actions of calcium/calmodulin-dependent protein kinases (CaMKs) and the calcium-regulated phosphatase Calcineurin, plays a crucial role in nervous system function, particularly in processes of behavioral plasticity that allow animals to adjust to environmental stimuli (Wayman et al., 2008; Baumgärtel et al., 2012). In *C. elegans* , CMK-1 (Calcium/Calmodulin-dependent protein kinase 1) and Calcineurin A/ TAX-6 regulate thermo-nociceptive behavior. These molecules are involved in modulating naïve responsiveness at 20°C, as well as in adaptation to repeated or persistent noxious heat stimulation (Schild et al., 2014; Lia and Glauser, 2020; Rudgalvyte et al., 2025; Rudgalvyte and Glauser, 2025). These roles are mediated through signaling in a partially overlapping set of neurons: CMK-1 functions in the AFD, ASER, and FLP thermosensory neurons, while Calcineurin A/ TAX-6 acts in the FLP neurons and in specific interneurons, including AVA and RIM (Schild et al., 2014; Rudgalvyte et al., 2025). Although complex antagonistic interactions between CMK-1 and TAX-6 signaling have been observed, the nature of their interplay within the FLP neurons remains unexplored.

In FLP neurons, the subcellular localization of CMK-1 is temperature-dependent and critical for modulating behavioral responsiveness. CMK-1 signaling in the cytoplasm promotes responsiveness, while nuclear CMK-1 signaling has an inhibitory effect (Schild et al., 2014). In wild-type animals grown at 20°C, CMK-1 accumulates in the cytoplasm. Within 90 minutes of exposure to noxious heat (28°C), CMK-1 progressively accumulates in the nucleus, contributing to thermal adaptation (Schild et al., 2014; Ippolito et al., 2021; Ippolito and Glauser, 2023). Given that Calcineurin A/ TAX-6 signaling inhibits behavioral adaptation (Rudgalvyte et al., 2025), we hypothesized that it might suppress the nuclear translocation of CMK-1 in FLP neurons upon sustained heat stimulation.

To test this, we quantified the nuclear-to-cytoplasmic signal ratio of a CMK-1 ::mNeonGreen fusion protein in FLP neurons of wild-type animals, *tax-6 ( jh107 )* gain-of-function mutants (Lee et al., 2004), and *tax-6 ( p675 )* loss-of-function mutants (Hedgecock and Russell, 1975; Kuhara et al., 2002). Our findings confirmed previous reports that (i) CMK-1 is cytoplasmic at 20°C and (ii) it translocates to the nucleus after a prolonged 90-min exposure to 28°C (Fig. 1). At 20°C, the *tax-6* -affecting mutations did not alter the localization of CMK-1 , which remained predominantly cytoplasmic. However, in *tax-6 ( jh107 )* animals with constitutive Calcineurin A/ TAX-6 activation, nuclear translocation at 28°C was completely blocked. Conversely, in *tax-6 ( p675 )* mutants lacking Calcineurin A/ TAX-6 activity, CMK-1 nuclear accumulation at 28°C was slightly enhanced. These results support our hypothesis that TAX-6 signaling counteracts CMK-1 nuclear translocation in response to noxious heat.

Together, these findings suggest a mechanism by which Calcineurin A/ TAX-6 might oppose thermo-nociceptive adaptation by preventing CMK-1 stimulus-dependent nuclear localization. Both reduced CMK-1 signaling in the nucleus and increased CMK-1 signaling in the cytoplasm have been shown to promote thermo-nociceptive responses (Schild et al., 2014). The precise mechanism by which Calcineurin A/ TAX-6 activity prevents CMK-1 nuclear accumulation remains to be determined. It could involve reduced nuclear import or enhanced nuclear export, as well as either direct or indirect effects of dephosphorylation events catalyzed by Calcineurin A/ TAX-6 , either within FLP neurons or in other tissues. Considering that noxious heat-evoked nuclear accumulation of CMK-1 is favored by its CKK-1-dependent phosphorylation on T179 (Ippolito and Glauser, 2023), the simplest model is that Calcineurin A/ TAX-6 counteracts CKK-1 by directly dephosphorylating this residue, thereby limiting CMK-1 nuclear accumulation. While attractive, this model remains speculative, as Calcineurin A/ TAX-6 could also influence CMK-1 localization through more indirect mechanisms.

A previous study showed that Calcineurin can regulate the localization of CaMKII at inhibitory synapses (Marsden et al., 2010). However, to our knowledge, our data provide the first evidence of a Calcineurin–CaMK interaction affecting the nuclear localization of a CaM kinase. Since both CaMKI/ CMK-1 and Calcineurin A/ TAX-6 are rapidly activated by calcium but act on different timescales and may respond to distinct calcium thresholds, we speculate that this inhibition could function as a negative feedback loop. Such a mechanism could ensure precise modulation of nuclear CaMK signaling in response to stimuli of varying intensity and duration. Given the strong evolutionary conservation and broad expression of CaMKs and Calcineurin, it would be interesting to explore whether similar regulatory mechanisms exist in other species and neuronal contexts.

## Methods

*Caenorhabditis elegans* were cultured under standard conditions on nematode growth medium (NGM) plates seeded with OP50 *Escherichia coli* at 20°C (Stiernagle, 2006). New strains were created by crossing each *tax-6* mutant with the DAG439 strain, which carries a single-copy *[mec-3p:: cmk-1 ::mNeonGreen]* transgene (previously described in Hostettler et al., 2017). First-day adult hermaphrodites were tested after a 90-min incubation at either 20°C or 28°C, as previously described (Ippolito et al., 2021). For imaging, animals were immobilized using sodium azide, and the nuclear-to-cytoplasmic fluorescence ratio in FLP cell bodies was measured by epifluorescence microscopy, as previously described (Ippolito et al., 2021). The data were acquired from independent experiments conducted over five different days. The number of scored neurons for each condition ( *n* ) is indicated in Figure 1. Statistical significance was assessed using a two-way ANOVA followed by Bonferroni post hoc tests.

## Reagents

**Table:** 

**Strain**	**Genotype**	**Available from**
PR675	*tax-6 ( p675 ) IV *	CGC
KJ306	*tax-6 ( jh107 ) IV *	CGC
DAG439	* domSi439[mec-3p:: cmk-1 ::mNeonGreen::3xFlag::unc-54UTR] II *	Glauser lab
DAG1371	* domSi439[mec-3p:: cmk-1 ::mNeonGreen::3xFlag::unc-54UTR] II ; tax-6 ( p675 ) IV *	Glauser lab
DAG1372	* domSi439[mec-3p:: cmk-1 ::mNeonGreen::3xFlag::unc-54UTR] II; tax-6 ( jh107 ) IV *	Glauser lab
